# Integrated multitissue analysis reveals distinct oxidative stress responses during recovery from cold shock in the Amazonian fish *Colossoma macropomum*

**DOI:** 10.1111/jfb.70604

**Published:** 2026-08-10

**Authors:** Naiara Melo, Isaac Konig, Stefania Priscilla de Souza, Maria Fernanda Aquino Freitas, Daniella Aparecida de Jesus Paula, Ronald Kennedy Luz, Priscila Vieira Rosa, Luis David Solis Murgas

**Affiliations:** ^1^ Faculty of Animal Science and Veterinary Medicine Federal University of Lavras, UFLA Lavras Minas Gerais Brazil; ^2^ Department of Biochemistry Federal University of Rio Grande do Sul (UFRGS) Porto Alegre Rio Grande do Sul Brazil; ^3^ Federal University of Piauí, Professor Cinobelina Elvas Campus Bom Jesus Piauí Brazil; ^4^ Departamento de Zootecnia, Laboratório de Aquacultura Universidade Federal de Minas Gerais Belo Horizonte Minas Gerais Brazil

**Keywords:** antioxidant enzymes, cold shock, integrated biomarker response (IBRv2), oxidative stress, Tambaqui

## Abstract

Tambaqui (*Colossoma macropomum*) is a tropical fish widely cultivated in South America. The increasing demand for this species has heightened interest in its cultivation in subtropical regions with greater thermal variability, where cold shock may induce oxidative stress. This study evaluated the effects of cold shock and subsequent recovery on antioxidant enzyme activity, which included superoxide dismutase (SOD), catalase (CAT), glutathione peroxidase (GPx), glutathione reductase (GR), glutathione S‐transferase (GST) and oxidative damage biomarkers (lipid peroxidation and protein carbonyl) in different tissues of juvenile tambaqui exposed to cold shock at 20°C for 24 h. Samples were collected before temperature reduction (baseline) and at 1, 24 and 48 h after returning to 28°C (recovery phase). Because sampling was restricted to the recovery period, the biomarkers primarily reflected post‐stressor physiological reorganization. Recovery responses were strongly tissue‐specific. The liver exhibited the broadest biomarker modulation, whereas the gills showed the highest integrated disturbance and the earliest response according to Integrated Biomarker Response version 2 (IBRv2). The brain displayed the clearest tendency towards restoration of basal redox homeostasis, while the intestine and muscle retained oxidative demands during later recovery. These findings demonstrate that post‐cold recovery in juvenile tambaqui is a dynamic and tissue‐dependent process and highlight the importance of multibiomarker and multitissue approaches for physiological monitoring and thermal stress assessment in tropical aquaculture species.

## 1 INTRODUCTION

Water temperature is one of the main limiting factors for fish because, as ectothermic animals, it determines the rate of biochemical reactions and, consequently, the energy balance available for functions such as feeding, digestion, locomotion, growth and reproduction (Reid et al., 2022; Volkoff & Rønnestad, 2020). In addition to the absolute value, the rate of thermal change is also crucial, as rapid changes tend to produce more severe effects than gradual ones, as they reduce the possibility of timely physiological adjustment (Donaldson et al., 2008; Reid et al., 2022). Thus, abrupt temperature drops over a short period characterize cold shock, which can trigger stress responses and physiological imbalances (e.g., changes in cortisol and glucose levels, ionic/acid–base disturbances, oxidative stress and increased maintenance costs). This is often accompanied by reduced locomotor performance and impaired reflexes/activity, with implications for vulnerability and mortality (Donaldson et al., 2008; Islam et al., 2021; Michie et al., 2020; Szekeres et al., 2014). This type of event can occur both under natural conditions and in anthropogenic situations, such as handling, transport and aquaculture systems with low thermal inertia (Donaldson et al., 2008; Reid et al., 2022; Szekeres et al., 2014).

Tambaqui (*Colossoma macropomum*) is one of the most important native species for inland aquaculture in South America, with production concentrated in tropical regions characterized by low annual thermal amplitude and typically warm waters (Agudelo et al., 2022; Hilsdorf et al., 2022; Valladão et al., 2018). Endemic to the Amazon and Orinoco basins, the species evolved in an environment driven by extreme seasonal fluctuations in dissolved oxygen and water chemistry (Val & Wood, 2022). To thrive in such heterogeneous habitats, *C. macropomum* exhibits remarkable phenotypic, biochemical and physiological plasticity (Val & Almeida‐Val, 2026). A classic example of its resilience is its high tolerance to severe hypoxia, characterized by the reversible expansion of the lower lip to facilitate aquatic surface respiration (Fagundes et al., 2026; Val & Wood, 2022). This morphological adaptation is supported by profound metabolic reprogramming, including the activation of the hypoxia‐inducible factor (HIF) pathway, downregulation of aerobic pathways and enhanced glycolytic flux to sustain local energy demands (Fagundes et al., 2026).

Although the species is recognized for its robustness in relation to some management stressors and these natural environmental extremes, it is a tropical fish with marked restrictions at low temperatures, which limits its performance and the feasibility of cultivation in subtropical regions (Agudelo et al., 2022). Thermal deviations from its optimal range strongly challenge its homeostasis and redox balance (Val & Wood, 2022), altering its antioxidant status and inducing severe cell damage (Amanajás et al., 2024). For instance, evidence from farming under subtropical conditions reports episodes of total mortality after consecutive days with minimum temperatures around 15°C (Meurer & Filho, 1997). Several studies indicate that water temperatures below approximately 15–18°C during the cold season constitute a limit for the establishment and expansion of the species, and that suboptimal ranges (below ~23°C) are associated with reduced performance (Woynárovich & Van Anrooy, 2019). In contrast, the range considered ideal/optimal for culture and performance is around 28–30°C, with experimental evidence of better growth and feed efficiency close to 30°C (Chu Koo et al., 2011; Hilsdorf et al., 2022; Woynárovich & Van Anrooy, 2019).

In addition to classical stress responses (endocrine and metabolic), rapid cooling and/or exposure to suboptimal temperatures can impact redox balance, as thermal changes alter mitochondrial function and the efficiency of aerobic metabolism, favouring the formation of reactive species and requiring adjustments in the antioxidant system (Lu et al., 2019; Reid et al., 2022; Tseng et al., 2011). Studies in teleosts, such as *Danio rerio*, demonstrate that cold shock can induce oxidative damage and tissue‐specific antioxidant responses, showing that different organs (e.g., liver, gills and brain) may display distinct patterns of lipid peroxidation, antioxidant enzyme expression/activity and apoptosis following acute cold exposure (Tseng et al., 2011; Wu et al., 2015).

For *C. macropomum*, studies have already shown that sudden reductions in temperature trigger haematological and metabolic alterations and modulate antioxidant components. However, much of the available knowledge still focuses on single‐tissue analyses and sampling restricted to the cold exposure period (dos Santos Silva et al., 2024; Paulino et al., 2026). For example, in juveniles subjected to a sudden decrease from 28 to 18°C, changes in plasma markers and liver antioxidant activities were reported within 1–24 h after the onset of the cold shock (dos Santos Silva et al., 2024). In parallel, studies with juvenile tambaqui reinforce that oxidative biomarkers and antioxidant defences are strongly tissue‐dependent, showing contrasting profiles among liver, intestine, gills, muscle, brain and plasma (Melo et al., 2024).

Considering these knowledge gaps, the present study aimed to expose juvenile *C. macropomum* to a rapid temperature decrease maintained for 24 h and to evaluate antioxidant responses and oxidative damage biomarkers in different tissues in the recovery phase. This approach allows the comparison of the magnitude and temporal dynamics of the responses, as well as the identification of which tissues are more responsive and which return more rapidly to baseline patterns.

## 2 MATERIALS AND METHODS

### 2.1 Study site and ethical approval

The present study was conducted in the laboratories of the Department of Animal Science at the Federal University of Lavras (UFLA), Brazil. All experimental procedures were approved by the Institutional Committee for the Ethical Use of Animals under protocol no. 29/17.

### 2.2 Experimental animals and rearing conditions

The animals used in this study were obtained from induced reproduction carried out at the Aquaculture Laboratory of the Federal University of Minas Gerais (LAQUA‐UFMG). Five‐day post‐hatch larvae were fed with *Artemia salina* for 15 days, followed by a powdered diet containing 46% crude protein up to 70 days post‐hatching (Santos et al., 2021). During this early phase, fish were reared in 28 L tanks connected to a Recirculating Aquaculture System (RAS) at a stocking density of 50 larvae L^−1^, with water temperature maintained at 28°C. Subsequently, the juveniles were transferred to 800 L tanks in a RAS, following routine maintenance protocols (Ananias et al., 2024; Santos et al., 2021). In this phase, the fish were kept at a density of 2000 individuals per tank at 28°C and fed with a powdered diet at 10% of their biomass, mixed with an extruded commercial feed (1.3–1.5 mm in diameter, Wean Prime line, Total Rações, Três Corações, Brazil) containing 45% crude protein.

Juvenile tambaqui (*C. macropomum*) with an initial body weight of 75.57 ± 7.13 g and a total length of 16.83 ± 0.92 cm were distributed among three 400 L tanks connected to a RAS equipped with mechanical and biological filtration, temperature control and supplemental aeration. Fish were acclimated to the experimental conditions for 15 days. The photoperiod was maintained at 12 h light:12 h dark (12L:12D) using a timer (Brasfort 8769). During acclimation, fish were kept under the following conditions: temperature of 28.12 ± 0.25°C, dissolved oxygen of 6.91 ± 0.27 mg L^−1^ and pH of 6.91 ± 0.27. Fish were fed three times daily with an extruded commercial diet (AQUOS Tropical; 2–4 mm; 320 g kg^−1^ crude protein, 7.5 g kg^−1^ lipids, 120 g kg^−1^ ash and 4.5 g kg^−1^ fibre). The animals were fasted for 24 h before the start of the experiment.

### 2.3 Cold shock protocol and recovery

Cold shock was induced by a controlled decrease in water temperature in the RAS from 28 to 20°C by water replacement using two auxiliary reservoirs with a combined volume of 600 L, consisting of one 400 L reservoir and one 200 L reservoir. The RAS consisted of three 400 L tanks containing fish. The water in the auxiliary reservoirs was cooled to 12°C using ice, which was added only to these reservoirs and not directly to the tanks containing fish, thereby preventing direct contact between ice and fish.

To induce the temperature decrease, the water outlet of the tanks was opened, allowing part of the 28°C water to leave the system while cooled water from the auxiliary reservoirs entered the RAS and mixed with the water in the tanks. Water movement during this procedure was driven by a Sarlo Aquafortis 20000 submersible pump, with a nominal flow capacity of 19,000 L h^−1^. Under the experimental configuration, the measured water flow was 22.0 L min^−1^ tank^−1^. Considering the three tanks, the total measured flow was 66.0 L min^−1^. The target temperature of 20°C was reached after 11 min, corresponding to an average cooling rate of 0.727°C min^−1^, equivalent to 43.6°C h^−1^. During the cooling procedure, continuous water circulation and supplemental aeration were maintained to promote water mixing and reduce thermal heterogeneity. Water temperature was monitored during the cooling process until the target temperature was reached.

Fish were maintained at 20°C for 24 h. After this period, the RAS temperature controller was reset to 28°C, allowing the water temperature to gradually return to the initial condition without abrupt heating. Recovery began after the 24 h exposure at 20°C with the adjustment of the thermal controller to 28°C. Temperature was gradually restored under continuous recirculation and supplemental aeration until reaching 28°C. Recovery time 0 h was defined as the moment when the water temperature reached 28°C, from which sampling was performed at 1, 24 and 48 h.

### 2.4 Sampling and tissue collection

Baseline sampling (0 h) was performed before the induction of thermal stress. After the system returned to 28°C (recovery phase), additional samplings were conducted at 1, 24 and 48 h. At each time point, six fish were collected per tank, totalling 18 fish per sampling point. Fish were euthanized by spinal cord section, followed by dissection and collection of brain, gills, intestine, liver and a portion of white muscle from the dorsal region. The tissues were immediately frozen in liquid nitrogen and stored at −80°C until sample processing. For statistical analyses, the tank was considered the experimental unit. Six fish were sampled from each tank at each time point (18 fish per time point in total), and fish from the same tank were treated as biological subsamples. Individual biomarker values were averaged within each tank, and the resulting tank mean (*n* = 3 tank means per time point) was used for all statistical analyses.

### 2.5 Tissue homogenization and protein quantification

Tissue samples were initially weighed using an analytical balance (Shimadzu AUW220D) and homogenized in ice‐cold saline buffer (0.138 M NaCl; 0.0027 M KCl; pH 7.2) at a ratio of 1:10 (w/v). The homogenates were centrifuged at 7000 × *g* for 15 min at 4°C (Multifuge X1R, Thermo Fisher Scientific, Waltham, MA, USA). The resulting supernatant was carefully collected, divided into four 500 μL aliquots and stored at −80°C until biochemical analyses.

Total protein concentration in the homogenates was determined following the Bradford method (Bradford, 1976). For this purpose, 10 μL of the supernatant was mixed with 200 μL of Bradford reagent, and a standard curve was constructed using bovine serum albumin as the reference protein. Absorbance was measured at 595 nm using a microplate reader. Protein concentrations were used to normalize the activities of antioxidant enzymes and the levels of oxidative damage biomarkers described below. All analyses were performed in triplicate, and the interval between tissue sampling and completion of the assays was approximately 1 week.

### 2.6 Antioxidant enzymes and oxidative damage biomarkers

Antioxidant enzyme activities were determined in tissue homogenates. Catalase (CAT, EC 1.11.1.6) activity was measured by monitoring the decomposition of hydrogen peroxide (H_2_O_2_) at 240 nm according to Aebi (1984). Superoxide dismutase (SOD, EC 1.15.1.1) activity was determined based on the inhibition of pyrogallol auto‐oxidation following the method of Madesh and Balasubramanian (1997), with absorbance measured at 570 nm. Glutathione peroxidase (GPx, EC 1.11.1.9) activity was quantified by monitoring NADPH oxidation at 340 nm in a coupled reaction system containing reduced glutathione (GSH) and glutathione reductase (GR), according to Günzler et al. (1984). GR (EC 1.8.1.7) activity was also determined by monitoring NADPH oxidation at 340 nm, following the method of Günzler et al. (1984). Glutathione S‐transferase (GST, EC 2.5.1.18) activity was measured at 340 nm using 1‐chloro‐2,4‐dinitrobenzene (CDNB) as a substrate according to Habig et al. (1974).

Oxidative damage was evaluated through lipid peroxidation and protein oxidation markers. Lipid peroxidation was estimated by measuring thiobarbituric acid reactive substances (TBARS), using malondialdehyde (MDA) as the reference compound, with absorbance read at 530 nm (Buege & Aust, 1978). Protein oxidation was assessed by determining protein carbonyl (PC) content after derivatization with 2,4‐dinitrophenylhydrazine (DNPH) according to Reznick and Packer (1994).

Detailed descriptions of the enzymatic assays and oxidative damage determinations are provided in the Supplementary Methods.

### 2.7 Integrated biomarker response version 2

Integrated biomarker response version 2 (IBRv2) was calculated according to the methodology proposed by Beliaeff and Burgeot (2002) and later refined by Sanchez et al. (2013). The index was calculated separately for each tissue, using the basal group as the reference (*X*_0*j*_). For each biomarker (*j*) and sampling time (*i*), the mean values (*X*_*ij*_) were normalized relative to the basal condition and expressed as a logarithmic ratio (*Y*_*ij*_ = log(*X*_*ij*_/*X*_0*j*_)). Subsequently, for each biomarker, the mean (*μ*_*j*_) and standard deviation (*s*_*j*_) of *Y*_*ij*_ were calculated considering all evaluated time points (0, 1, 24 and 48 h), and the values were standardized using a *z*‐score transformation (Z_*ij*_ = (*Y*_*ij*_ − *μ*_*j*_)/*s*_*j*_). The deviation relative to the reference group was then obtained as *A*_*ij*_ = Z_*ij*_ − *Z*_0*j*_. The IBRv2 for each time point was calculated as the sum of the absolute values of these deviations (IBRv2_*i*_ = Σ_*j*_|A_*ij*_|). The A_*ij*_ values were represented using star plots, in which positive and negative values indicate induction or inhibition of each biomarker relative to the basal condition, respectively. Finally, the integrated index for each tissue throughout the experiment was obtained by summing the IBRv2 values across all experimental time points.

### 2.8 Statistical analysis

Statistical analyses were performed using Minitab® 23 (Minitab LLC, State College, PA, USA), and graphs were generated using Prism version 10 (GraphPad Software, San Diego, CA, USA). The tank was considered the experimental unit, with each value corresponding to the mean of one tank. Data normality was assessed using the Shapiro–Wilk test, and homogeneity of variances was evaluated using Levene's test. When necessary, data were transformed using the Box‐Cox procedure to meet the assumptions of parametric analysis. After transformation, normality and homogeneity of variances were reassessed. For each tissue, differences among experimental time points were analysed using one‐way ANOVA. When significant effects were detected, pairwise comparisons were conducted using Tukey's post hoc test. The level of significance was set at 5%. Results are expressed as mean ± standard deviation using untransformed data for biological interpretation.

## 3 RESULTS

### 3.1 Antioxidant enzymes and oxidative damage biomarkers

During the experimental period, no mortality was observed. The results of the antioxidant enzymes SOD, CAT, GPx, GR and GST in the different tissues are presented in Figure 1a–e. The oxidative damage biomarkers, mMDA and protein carbonyls (PCs), are shown in Figures 2a–e and 3a–e, respectively.

**FIGURE 1:**
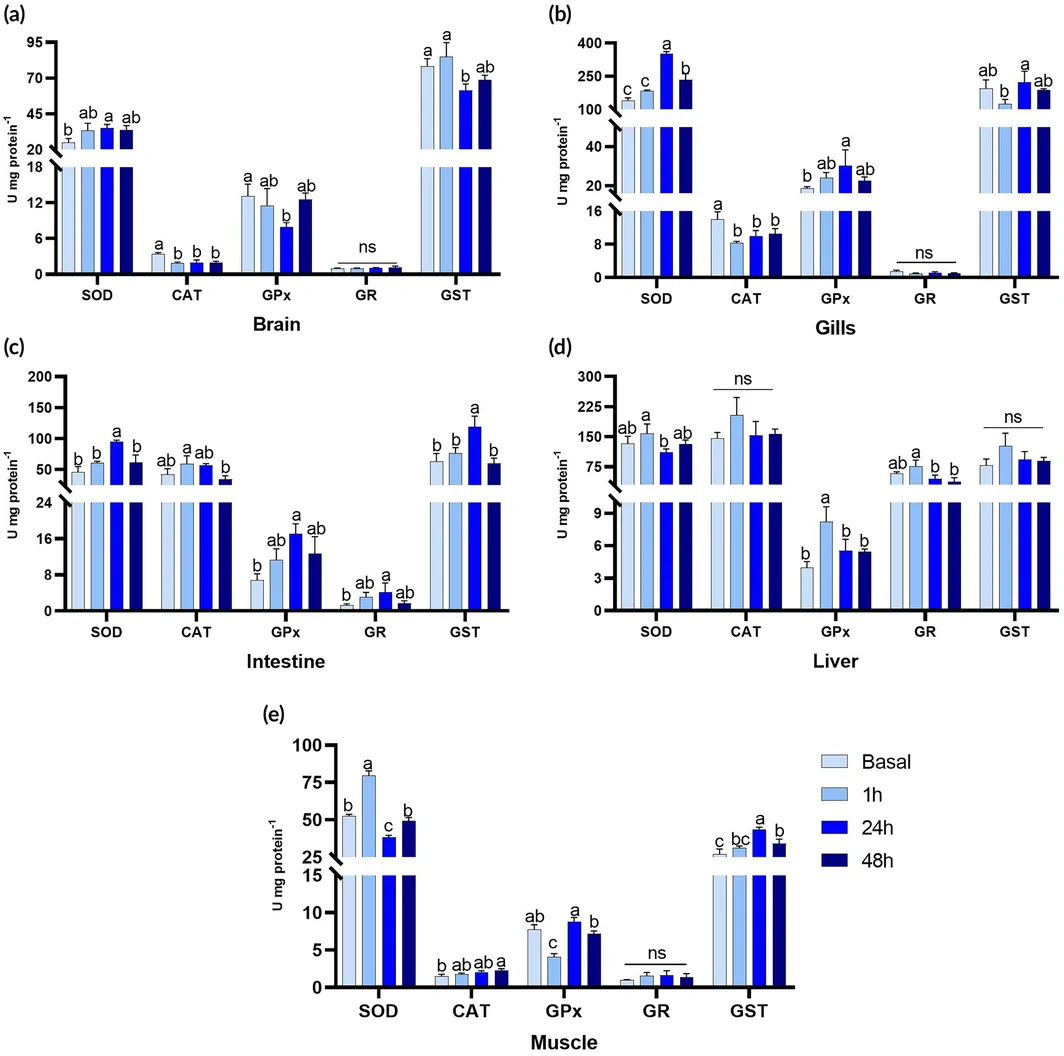
Antioxidant enzyme activities in different tissues of juvenile tambaqui (*Colossoma macropomum*) subjected to cold shock (20°C for 24 h). Samples were collected before temperature reduction (baseline) and at 1, 24 and 48 h after the water temperature returned to 28°C (recovery phase). Panels show the activities of superoxide dismutase (SOD), catalase (CAT), glutathione peroxidase (GPx), glutathione reductase (GR) and glutathione S‐transferase (GST) in the brain (a), gills (b), intestine (c), liver (d) and muscle (e). Data are expressed as mean ± standard deviation (SD). Statistical analyses (ANOVA) were performed using tank means as the experimental units (*n* = 3 tanks per sampling time, with 6 fish sampled from each tank; 18 fish in total per sampling time). Different lowercase letters denote significant differences among sampling times within the same tissue according to Tukey's post hoc test (*p* < 0.05), whereas ns indicates no significant differences.

**FIGURE 2:**
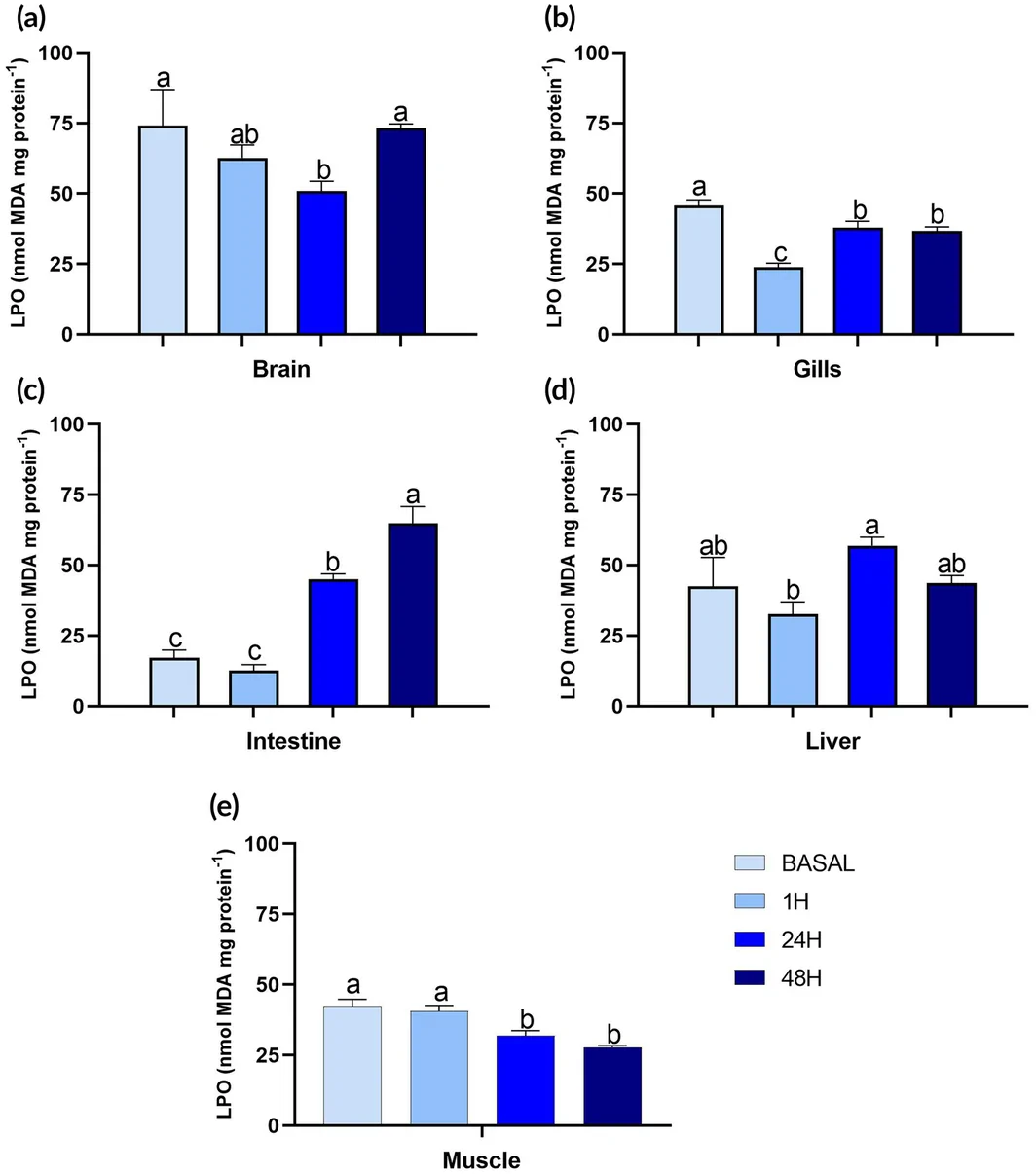
Lipid peroxidation (LPO) levels measured as malondialdehyde (MDA) by the thiobarbituric acid reactive substances (TBARS) assay in tissues of juvenile tambaqui (*Colossoma macropomum*) exposed to cold shock (20°C for 24 h). Sampling was performed before temperature reduction (baseline) and at 1, 24 and 48 h after the return to 28°C (recovery phase). Panels show MDA levels over time in the brain (a), gills (b), intestine (c), liver (d) and muscle (e). Data are expressed as mean ± standard deviation (SD). Statistical analyses (ANOVA) were performed using tank means as the experimental units (*n* = 3 tanks per sampling time, with 6 fish sampled from each tank; 18 fish in total per sampling time). Different lowercase letters denote significant differences among sampling times within the same tissue according to Tukey's post hoc test (*p* < 0.05).

**FIGURE 3:**
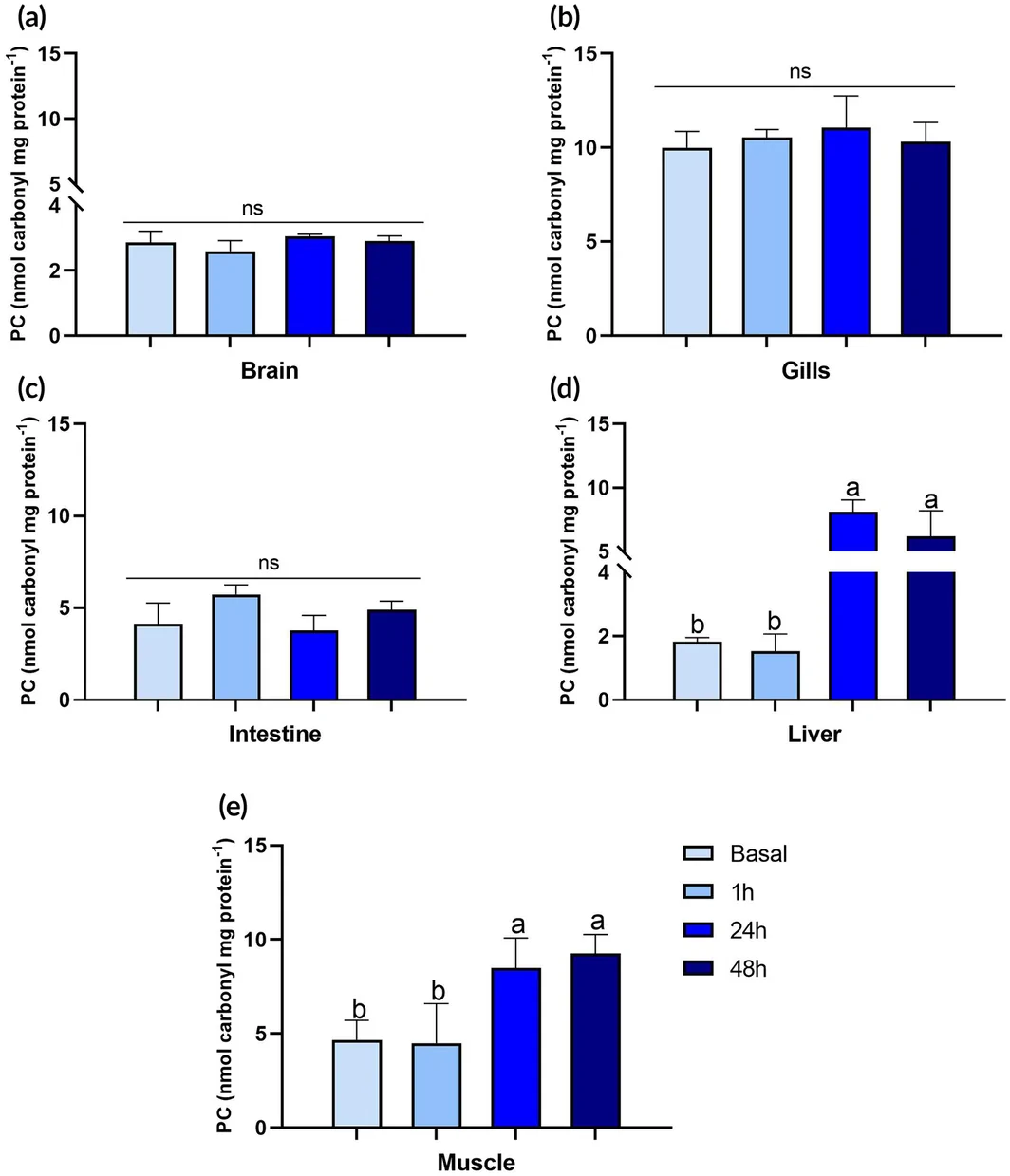
Protein oxidation assessed by protein carbonyl (PC) content in tissues of juvenile tambaqui (*Colossoma macropomum*) following cold shock (20°C for 24 h). Samples were obtained before temperature reduction (baseline) and at 1, 24 and 48 h after the temperature returned to 28°C (recovery phase). Panels present PC levels over time in the brain (a), gills (b), intestine (c), liver (d) and muscle (e). Data are expressed as mean ± standard deviation (SD). Statistical analyses (ANOVA) were performed using tank means as the experimental units (*n* = 3 tanks per sampling time, with 6 fish sampled from each tank; 18 fish in total per sampling time). Different lowercase letters denote significant differences among sampling times within the same tissue according to Tukey's post hoc test (*p* < 0.05), whereas ns indicates no significant differences.

In the brain (Figure 1a), a significant effect of time was observed on the activities of SOD (*p* = 0.030), CAT (*p* = 0.002), GPx (*p* = 0.039) and GST (*p* = 0.008), whereas GR did not vary significantly (*p* = 0.229). SOD activity (*p* = 0.030) was higher at 24 h compared with the baseline. CAT activity (*p* = 0.002) decreased at 1 h and remained lower than the baseline at both 24 and 48 h. GPx activity (*p* = 0.039) showed a reduction at 24 h relative to the baseline. GST activity (*p* = 0.008) was lower at 24 h, with intermediate values observed at 48 h (Figure 1a). MDA levels in the brain (Figure 2a) varied over time (*p* = 0.012), showing a reduction at 24 h compared with the baseline and 48 h. In contrast, PC levels did not show significant differences over time (*p* = 0.242) (Figure 3a).

In the gills (Figure 1b), a significant effect of time was observed on SOD (*p* < 0.001), CAT (*p* = 0.004), GPx (*p* = 0.025) and GST (*p* = 0.035), whereas GR did not differ among time points (*p* = 0.067). SOD activity (*p* < 0.001) increased markedly at 24 h and remained elevated at 48 h compared with the baseline and 1 h. CAT activity (*p* = 0.004) decreased at 1 h and remained lower than the baseline at 24 and 48 h. GPx activity (*p* = 0.025) was higher at 24 h compared with the baseline. GST activity (*p* = 0.035) increased at 24 h relative to 1 h. MDA levels in the gills (Figure 2b) were also affected (*p* < 0.001), showing a reduction at 1 h and remaining lower than the baseline at 24 and 48 h. In contrast, PC levels did not vary significantly over time (*p* = 0.679; Figure 3b).

In the intestine (Figure 1c), time significantly affected the activities of SOD (*p* < 0.001), CAT (*p* = 0.025), GPx (*p* = 0.009), GR (*p* = 0.034) and GST (*p* = 0.001). SOD activity (*p* < 0.001) peaked at 24 h, showing higher values than the baseline, 1 and 48 h. CAT activity (*p* = 0.025) was higher at 1 h compared with 48 h, while the baseline and 24 h showed intermediate levels. GPx (*p* = 0.009) and GR (*p* = 0.034) activities were higher at 24 h relative to the baseline. GST activity (*p* = 0.001) was also higher at 24 h compared with the other time points. MDA levels in the intestine (Figure 2c) varied markedly over time (*p* < 0.001), increasing at 24 h and reaching the highest value at 48 h. In contrast, PC levels did not differ significantly among time points (*p* = 0.060; Figure 3c).

In the liver (Figure 1d), time significantly affected the activities of SOD (*p* = 0.048), GPx (*p* = 0.003) and GR (*p* = 0.010), whereas CAT (*p* = 0.188) and GST (*p* = 0.098) did not show significant variation (Figure 1d). SOD activity (*p* = 0.048) was higher at 1 h and lower at 24 h. GPx activity (*p* = 0.003) increased at 1 h compared with the other time points. GR activity (*p* = 0.010) was also higher at 1 h, followed by a reduction at 24 and 48 h. MDA levels in the liver (Figure 2d) varied over time (*p* = 0.007), showing an increase at 24 h compared with 1 h. In addition, PC content increased markedly at 24 and 48 h (*p* < 0.001; Figure 3d) relative to the baseline and 1 h.

In the muscle (Figure 1e), time significantly affected the activities of SOD (*p* < 0.001), CAT (*p* = 0.014), GPx (*p* < 0.001) and GST (*p* < 0.001), whereas GR did not show significant variation (*p* = 0.388). SOD activity (*p* < 0.001) was higher at 1 h and reached the lowest value at 24 h, returning to a level similar to the baseline at 48 h. CAT activity (*p* = 0.014) was higher at 48 h compared with the baseline (Figure 1e). GPx activity (*p* < 0.001) decreased at 1 h and increased at 24 h (highest value), with 48 h showing intermediate levels. GST activity (*p* < 0.001) increased at 24 h and remained elevated at 48 h. MDA levels in the muscle (Figure 2e) were significantly affected (*p* < 0.001), showing reductions at 24 and 48 h compared with the baseline and 1 h. PC content, on the other hand, increased at 24 and 48 h (*p* = 0.006) (Figure 3e) relative to the baseline and 1 h.

### 3.2 Integrated biomarker response version 2

IBRv2, considering the biomarkers evaluated in the different tissues, is presented in Figure 4. Overall, the highest total IBRv2 (ΣIBRv2) was observed in the gills (30.41; Figure 4c), followed by muscle (27.16; Figure 4i), brain (26.93; Figure 4a) and intestine (26.57; Figure 4e), whereas the liver showed the lowest ΣIBRv2 (23.03; Figure 4g).

**FIGURE 4:**
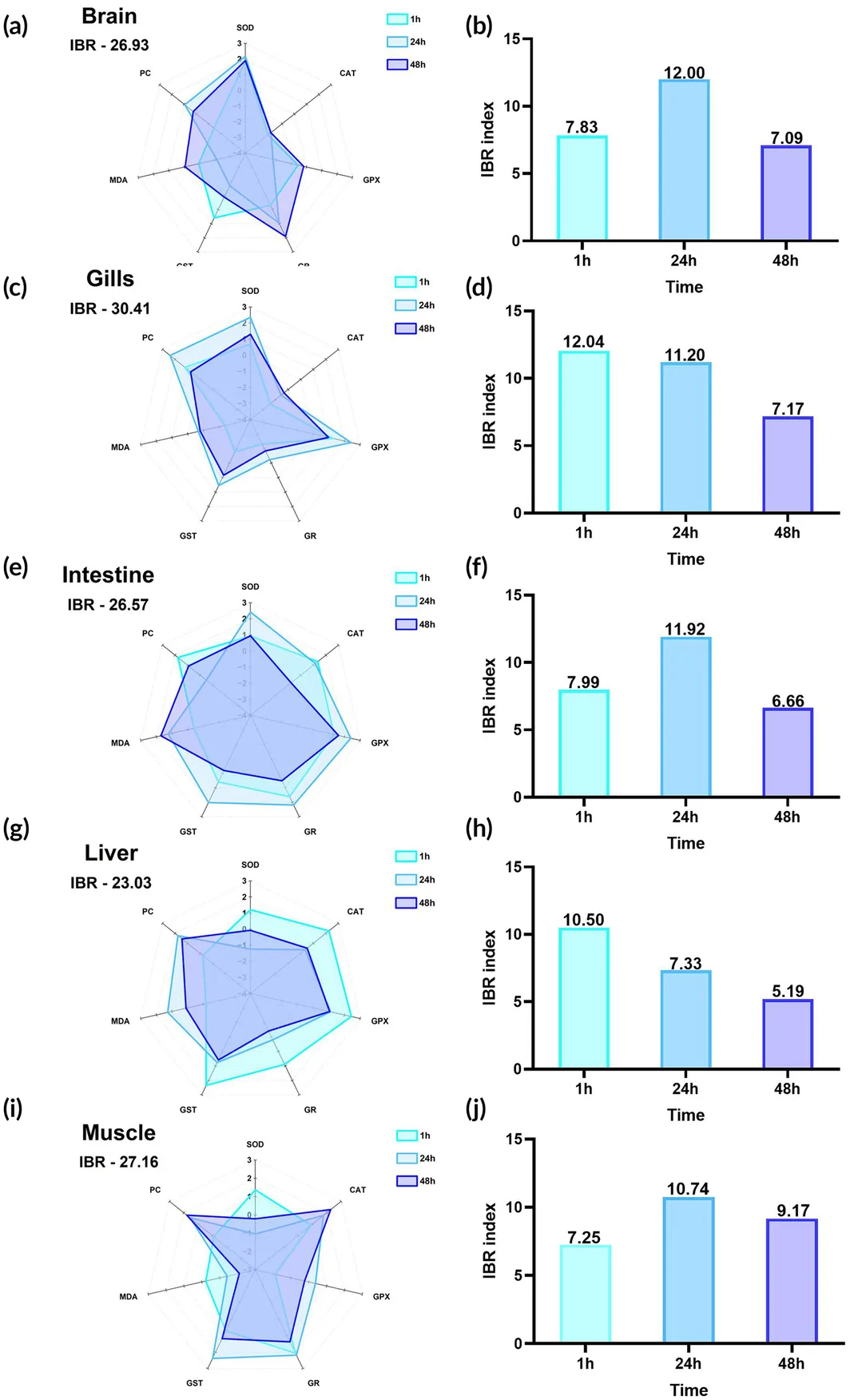
Integrated biomarker response (IBRv2) in tissues of juvenile tambaqui (*Colossoma macropomum*) subjected to cold shock (20°C for 24 h). Samples were collected before temperature reduction (baseline) and at 1, 24 and 48 h after the return to 28°C (recovery phase). Radar plots illustrate the IBRv2 profiles and the overall IBRv2 index over time in the brain (a, b), gills (c, d), intestine (e, f), liver (g, h) and muscle (i, j). IBRv2 was calculated using tank‐level means (*n* = 3 tanks per sampling time, with 6 fish sampled from each tank; 18 fish in total per sampling time).

Regarding the temporal variation of the index (Figure 4b,d,f,h,j), the brain showed a peak in the integrated response at 24 h (IBRv2 = 12.00) compared with 1 h (7.83) and 48 h (7.09) (Figure 4b). In the gills, the highest value occurred at 1 h (IBRv2 = 12.04), remaining elevated at 24 h (11.20) and decreasing at 48 h (7.17) (Figure 4d). In the intestine, IBRv2 also reached its highest value at 24 h (11.92), compared with 1 h (7.99) and 48 h (6.66) (Figure 4f). In the liver, the highest IBRv2 was observed at 1 h (10.50), followed by a progressive decrease at 24 h (7.33) and 48 h (5.19) (Figure 4h). In the muscle, IBRv2 was highest at 24 h (10.74) and remained relatively elevated at 48 h (9.17) compared with 1 h (7.25) (Figure 4j).

## 4 DISCUSSION

In fish, thermal changes can destabilize redox balance and promote reactive oxygen species (ROS) formation, so the activation of antioxidant defences is a central component for maintaining homeostasis and mitigating damage to lipids and proteins (Lushchak, 2011; Reid et al., 2022). A key aspect of our experimental design is that sampling was conducted exclusively during the recovery phase, so the analysed biomarkers primarily reflect physiological reorganization after cold shock and the redox costs associated with metabolic resumption. This is relevant because recovery can be biologically critical and not necessarily milder than exposure to the stressor, and redox challenges may persist even after thermal restoration (Sánchez‐Nuño et al., 2018). From an applied perspective, understanding the recovery dynamics after cold shock in *C. macropomum*, a tropical species with limitations to low temperatures, can support thermal management strategies and physiological monitoring aimed at reducing sublethal damage, mortality and production losses in aquaculture.

The cerebral response to cold shock results from the integrated action of different antioxidant systems (Wu et al., 2015). SOD, which constitutes the first line of defence, and GR maintained stable activities in the brain, suggesting preservation and maintenance of thiol redox status. This interpretation is consistent with studies in *D. rerio* and *Carassius auratus*, where GSH metabolism in brain tissue was preserved even under acute cold exposure (Lushchak & Bagnyukova, 2006a; Tseng et al., 2011). Additionally, the early mobilization of GST at 1 h suggests an immediate response in the detoxification of secondary oxidation products (Lushchak & Bagnyukova, 2006b). An efficient antioxidant system is important since this organ has high metabolic demand and elevated levels of polyunsaturated fatty acids, making it naturally vulnerable to ROS (Papoutsoglou, 2012; Ratko et al., 2022). The effectiveness of the antioxidant defence is confirmed by the absence of protein carbonylation, corroborating findings in *Astyanax lacustris* (Ratko et al., 2022). In the literature, this protection of the neuronal proteome has been attributed to the induction of heat shock proteins, which act as molecular chaperones, preventing protein misfolding and oxidation (Li et al., 2024). In parallel, the reduction in MDA levels at 24 h suggests the neutralization of peroxides, metabolically supported by the pentose phosphate pathway, whose activation of the enzyme G6PDH provides NADPH essential for GSH recycling, protecting membranes and preventing apoptosis (Ratko et al., 2022; Toni et al., 2019).

The integration of all these biomarkers through the IBRv2 index allows visualization of the overall adaptive effort of brain tissue during recovery. The expansion of the area occupied by the index at 24 h characterizes what the literature defines as the physiological reorganization window, representing the moment of greatest mobilization to achieve a new homeostatic state (Campos et al., 2019; Joy et al., 2017). Mathematically, this peak at 24 h is driven by specific variables that act as vectors within the index (Beliaeff & Burgeot, 2002), with the activity of glutathione system enzymes, together with the reversal of MDA levels, being the main factors expanding the radar chart area at this time point. The decline toward baseline levels at 48 h indicates that the brain established effective compensatory mechanisms, with adaptive plasticity and recovery capacity after cold shock, without compromising the functional integrity of the organ. This reflects a maximal physiological mobilization effort to neutralize ROS before the onset of permanent damage (Campos et al., 2019; Li et al., 2024).

In cultured tropical species such as *C. macropomum*, cold stress can rapidly compromise gill function, as this tissue acts as the primary interface with the environment and is highly susceptible to structural alterations and redox imbalance induced by low temperatures (Refaey et al., 2023; Schleger et al., 2024; Wen et al., 2018). In our study, the recovery phase was accompanied by a biphasic antioxidant response in this tissue. In the first hour of recovery, reduced CAT and GST activities were observed, while SOD activity remained unchanged, suggesting a lower capacity for peroxide removal, consistent with the transient modulation of antioxidant enzymes already described for gills under acute thermal challenges (Lushchak & Bagnyukova, 2006a; Rossi et al., 2017). In subsequent time points, increased activities of SOD, GPx and GST were observed, while CAT remained reduced and GR activity showed no change, suggesting a greater contribution of the glutathione‐dependent antioxidant system. This pattern is consistent with the literature, which describes increased participation of these enzymes during recovery after thermal challenges (da Silva et al., 2024; Schleger et al., 2024; Wen et al., 2018).

The maintenance of PC levels in the gills, together with the reduction in MDA levels, is consistent with the limitation of oxidative damage in gill tissue throughout recovery. Overall, the data suggest that although the initial phase of recovery involves modulation of enzymes associated with peroxide removal, *C. macropomum* exhibits antioxidant adjustment capacity in the gills, favouring the reorganization of the redox response during recovery (Duan et al., 2024). The gill IBRv2 index complements these findings by highlighting the overall adaptive effort of the organ through a rapid response in the first hour of recovery, associated with initial changes in CAT and GST and a reduction in MDA (Devin et al., 2014). At 24 h, the maintenance of the index indicates persistence of the adaptive effort, associated with increased SOD, GPx and GST, reflecting biochemical adjustments aimed at stabilizing redox status (Wen et al., 2018). Finally, the reduction of the index at 48 h indicates progressive stabilization and return to homeostasis, suggesting that compensatory mechanisms were effective in restoring gill function and preserving the functional integrity of the fish after cold shock (Campos et al., 2019; Schleger et al., 2024).

Although the intestine is often addressed in feeding trials and studies of nutritional stress, this tissue also deserves attention under cold stress conditions, as it plays a central role in nutrient digestion and absorption and acts as a physico‐immunological barrier (Guerreiro et al., 2023; Ramos‐Oliveira et al., 2026). This functional combination results in high metabolic activity of the epithelium, making the intestine particularly sensitive to changes in redox balance (Chen et al., 2023; Chu et al., 2025; Liu et al., 2025). In our study, we observed a compensatory antioxidant response within the first 24 h, evidenced by increased activities of SOD, GPx, GST and GR, while the maintenance and subsequent reduction of CAT activity suggest a greater contribution of the glutathione‐dependent antioxidant system in peroxide removal (Lv et al., 2021). This pattern is consistent with that described in teleost species such as *Brachymystax lenok tsinlingensis* and *D. rerio* (Chu et al., 2025; Fang et al., 2023).

Regarding oxidative damage in the intestine, no changes were observed in PC levels. In contrast, lipid peroxidation increased at 24 h, peaking at 48 h, indicating that the antioxidant response was not sufficient to prevent the progression of oxidative damage. The high content of polyunsaturated fatty acids in intestinal cells, resulting from lipid absorption, makes this tissue particularly susceptible to oxidation (Chen et al., 2023; Fu et al., 2025; Li et al., 2023; Liu et al., 2025; Magouz et al., 2022). This pattern is consistent with reports in teleosts exposed to acute stressors, showing increased intestinal MDA levels (Chen et al., 2023; Dawood et al., 2022; Fu et al., 2025; Li et al., 2021; Zhou et al., 2022). Complementing these findings, the IBRv2 index identified a peak in physiological reorganization at 24 h and a decline at 48 h, indicating that, despite the delayed peak in MDA levels, the overall set of compensatory mechanisms was effective in stabilizing homeostasis (Lv et al., 2021).

In studies investigating different types of stress in fish, the liver is among the main tissues evaluated due to its central role in metabolism, biotransformation and maintenance of homeostasis (Figueiredo‐Fernandes et al., 2006). This high energetic demand makes hepatic tissue particularly susceptible to ROS production under thermal challenge conditions (Sun et al., 2020; Wang et al., 2019). In our study, during the recovery phase after cold stress, the liver of *C. macropomum* showed increased activities of SOD, GPx and GR within the first hour of recovery. This pattern is consistent with a higher oxidative demand in hepatic tissue during post‐cold physiological reorganization, a condition that, in other studies, has been associated with intensified oxidative metabolism and electron leakage in the respiratory chain (Brand, 2016; Mailloux, 2018; Qi et al., 2013).

In this context, the absence of an increase in MDA in the liver compared to baseline is consistent with an initial limitation of oxidative damage to lipids, a pattern also described in juveniles of *C. macropomum* subjected to thermal challenges (dos Santos Silva et al., 2024). On the other hand, the maintenance of the hepatic antioxidant response throughout recovery appears to depend, in part, on the activity of GPx and GR and on the supply of NADPH required for the regeneration of GSH from GSSG (Fan et al., 2014). In other species, the literature indicates that, under acute thermal challenges, this demand may be associated with the involvement of the pentose phosphate pathway and G6PDH (Dalvi et al., 2017; Guillen et al., 2019; Laiz‐Carrión et al., 2002). The reduction in SOD, GPx and GR activities, together with increased protein carbonylation, indicates diminished hepatic antioxidant capacity at later stages and enhanced protein damage, consistent with studies showing that proteins are preferential targets under thermal stress (Grim et al., 2010; Malek et al., 2004; Wen et al., 2018). Unlike the pattern observed in the IBRv2 in other tissues, the liver exhibited an immediate response followed by a progressive decline in subsequent hours (Campos et al., 2019; Devin et al., 2014). In this specific context of hepatic tissue, this reduction does not indicate homeostatic recovery, but rather a possible limitation in sustaining the antioxidant response over time, suggesting greater susceptibility to the progression of structural oxidative damage (da Silva et al., 2024; Wang et al., 2019). This behaviour corroborates studies identifying the liver as a highly demanding organ under thermal stress, in which prolonged exposure may compromise the antioxidant system and affect cellular integrity (Rossi et al., 2017; Roychowdhury et al., 2021; Sun et al., 2020).

Skeletal muscle represents the largest fraction of fish body mass, constitutes a functionally diverse tissue, and responds sensitively to metabolic and environmental changes, with direct implications for growth, performance and final product quality (Chu et al., 2021; Dettleff et al., 2020; Johnston et al., 2011). In this context, cold stress imposes significant physiological and metabolic challenges on teleost muscle tissue (Guderley, 2004). During the recovery phase, we observed modulation of the antioxidant response in this tissue throughout the experimental period, with changes in enzymatic activities but no evidence of a sustained protective antioxidant response. In contrast to the enzymatic profile, muscle showed reduced lipid peroxidation (MDA), suggesting stability of the lipid component, possibly associated with membrane remodelling and reduced propagation of peroxidation at low temperatures (Chu et al., 2021; Grim et al., 2010; Malek et al., 2004). On the other hand, there was a progressive increase in protein carbonylation, indicating a greater incidence of oxidative damage to proteins during recovery, a pattern also described in other species (Pereira et al., 2023).

Overall, the data indicate a dissociation between MDA and PC, with greater vulnerability of the protein component in muscle. This pattern is represented by the IBRv2 as a scenario of metabolic effort and vulnerability. Muscle reached its peak mobilization at 24 h, maintaining a high plateau up to 48 h. The expansion and maintenance of the radar chart area illustrate the continuous effort of the antioxidant system in response to the challenge imposed by recovery after cold shock (Campos et al., 2019). However, consistent with the dissociation observed in damage biomarkers, the persistence of this elevated index indicates that the muscle response capacity was directed and limited (Grim et al., 2010; Kammer et al., 2011). Although mobilization was effective in protecting membrane lipid structures, it was not sufficient to fully neutralize ROS, allowing the progression of oxidative stress and leading to protein carbonylation (Corrêa et al., 2024; Melo et al., 2024).

In the context of tropical aquaculture species, cold shock episodes represent critical events, as they may compromise physiological functions even after thermal restoration. In *C. macropomum*, recovery after cold shock was tissue‐specific, with a temporal redistribution of redox costs among organs: gills responded more promptly, the liver concentrated the integrated response in the early phases, while brain and intestine expressed a relevant part of this reorganization at later time points, and muscle showed a slower recovery. This pattern is consistent with the tissue variability described in juvenile tambaqui and in other species subjected to different stressors (Corrêa et al., 2024; Melo et al., 2024) and reinforces IBRv2 as a useful tool for integrating complex redox responses.

## 5 CONCLUSION

The present study demonstrates that post‐cold recovery in juvenile *C. macropomum* is a dynamic, time‐dependent and tissue‐specific process. The liver stood out for the greatest amplitude of biomarker changes, suggesting high redox reactivity and lower sustainability of the antioxidant response throughout recovery. In parallel, the gills responded immediately, while the muscle and intestine showed more delayed oxidative damage. The brain, in turn, showed a greater tendency to restore basal homeostasis. The multitissue approach, combined with IBRv2, allowed the integration of antioxidant responses across different tissues, revealing that recovery involves biological costs even after thermal restoration. These findings highlight the importance of incorporating the recovery phase into experimental assessments of cold stress in tropical fish and provide a physiological basis for management and monitoring strategies applied to aquaculture.

## AUTHOR CONTRIBUTIONS

**Naiara Melo:** Data collection and analysis; writing the first draft. **Isaac Konig:** Data collection and analysis; writing the first draft. **Stefania Priscilla de Souza:** Data collection. **Maria Fernanda Aquino Freitas:** Data collection. **Daniella Aparecida de Jesus Paula:** Data collection. **Ronald Kennedy Luz:** Funding; supervision; conceptualization; review and editing of the final draft. **Priscila Vieira Rosa:** Funding; supervision; conceptualization; review and editing of the final draft. **Luis David Solis Murgas:** Funding; supervision; conceptualization; review and editing of the final draft. All authors have read and approved the final version of this manuscript.

## FUNDING INFORMATION

The authors acknowledge the Conselho Nacional de Desenvolvimento Científico e Tecnológico (CNPq, Brazil) for financial support (152905/2024‐3 and 404911/2024‐2 to Naiara Melo; 310170/2023‐0 to Ronald Kennedy Luz; 403631/2024‐6 and 310149/2023‐1 to Priscila Vieira Rosa; and 310324/2023‐8 to Luis David Solis Murgas).

## CONFLICT OF INTEREST STATEMENT

The authors declare no conflicts of interest.

## Supporting information


**Data S1:** Supplemental methods.

## Data Availability

The data that support the findings of this study are available on request from the corresponding author.

## References

[jfb70604-bib-0001] Aebi, H. (1984). Catalase in vitro. Methods in Enzymology, 105, 121–126. 10.1016/s0076-6879(84)05016-3 6727660

[jfb70604-bib-0002] Agudelo, J. F. G. , Mastrochirico‐Filho, V. A. , Borges, C. H. d. S. , Ariede, R. B. , Lira, L. V. G. , Neto, R. R. d. O. , de Freitas, M. V. , Sucerquia, G. A. L. , Vera, M. , Berrocal, M. H. M. , & Hashimoto, D. T. (2022). Genomic selection signatures in farmed *Colossoma macropomum* from tropical and subtropical regions in South America. Evolutionary Applications, 15, 679–693. 10.1111/eva.13351 PMC9046916 35505878

[jfb70604-bib-0003] Amanajás, R. D. , Mota da Silva, J. , de Nazaré Paula da Silva, M. , & Val, A. L. (2024). Temperature alters antioxidant status and induces cell damage in the Amazonian fish tambaqui. Journal of Thermal Biology, 126, 103995. 10.1016/j.jtherbio.2024.103995 39616715

[jfb70604-bib-0004] Ananias, I. d. M. C. , Silva, S. d. S. , dos Santos, F. A. C. , Souza, A. d. S. , Magalhães, T. B. , Reis, P. A. R. , Favero, G. C. , & Luz, R. K. (2024). Tambaqui production at different stocking densities in RAS: Growth and physiology. Fishes, 9, 19. 10.3390/fishes9010019

[jfb70604-bib-0005] Beliaeff, B. , & Burgeot, T. (2002). Integrated biomarker response: A useful tool for ecological risk assessment. Environmental Toxicology and Chemistry, 21, 1316–1322. 10.1002/etc.5620210629 12069320

[jfb70604-bib-0006] Bradford, M. M. (1976). A rapid and sensitive method for the quantitation of microgram quantities of protein utilizing the principle of protein–dye binding. Analytical Biochemistry, 72, 248–254. 10.1006/abio.1976.9999 942051

[jfb70604-bib-0007] Brand, M. D. (2016). Mitochondrial generation of superoxide and hydrogen peroxide as the source of mitochondrial redox signaling. Free Radical Biology and Medicine, 100, 14–31. 10.1016/j.freeradbiomed.2016.04.001 27085844

[jfb70604-bib-0008] Buege, J. A. , & Aust, S. D. (1978). Microsomal lipid peroxidation. Methods in Enzymology, 52, 302–310. 10.1016/s0076-6879(78)52032-6 672633

[jfb70604-bib-0009] Campos, D. F. , Braz‐Mota, S. , Val, A. L. , & Almeida‐Val, V. M. F. (2019). Predicting thermal sensitivity of three Amazon fishes exposed to climate change scenarios. Ecological Indicators, 101, 533–540. 10.1016/j.ecolind.2019.01.051

[jfb70604-bib-0010] Chen, J. , Xu, P. , Wen, H. , Xue, M. , Wang, Q. , He, J. , He, C. , Su, S. , Li, J. , Yu, F. , Feng, W. , Wang, M. , Song, C. , & Li, H. (2023). Hypothermia‐mediated oxidative stress induces immunosuppression, morphological impairment and cell fate disorder in the intestine of freshwater drum, *Aplodinotus grunniens* . Aquaculture, 575, 739805. 10.1016/j.aquaculture.2023.739805

[jfb70604-bib-0011] Chu Koo, F. , Stewart, P. , Babilonia, J. L. , Garcia‐Davilia, C. , & Kohler, C. (2011). Water temperature effects on growth, feed utilization and survival of black pacu (*Colossoma macropomum*) fingerlings. Folia Amazonica, 20, 15–21. 10.24841/fa.v20i1-2.359

[jfb70604-bib-0012] Chu, P. , Li, Y. , Han, X. , Li, X. , Liu, Y. , Tang, Z. , Yin, S. , & Wang, T. (2025). Mechanism of low temperature‐induced intestinal damage in *Danio rerio* and the mitigating effect of alanylglutamine. Water Biology and Security, 4, 100377. 10.1016/j.watbs.2025.100377

[jfb70604-bib-0013] Chu, P. , Wang, T. , Sun, Y. R. , Chu, M. X. , Wang, H. Y. , Zheng, X. , & Yin, S. (2021). Effect of cold stress on the MAPK pathway and lipidomics on muscle of *Takifugu fasciatus* . Aquaculture, 540, 736691. 10.1016/j.aquaculture.2021.736691

[jfb70604-bib-0014] Corrêa, A. P. N. , da Silva, N. G. , Ratko, J. , da Silva, D. O. , Schleger, I. C. , Pereira, D. M. C. , Neundorf, A. K. A. , de Souza, M. R. D. P. , Herrerias, T. , & Donatti, L. (2024). Influence of acute heat shock on antioxidant defense of tropical fish, *Psalidodon bifasciatus* . Comparative Biochemistry and Physiology, Part B, 274, 111006. 10.1016/j.cbpb.2024.111006 38977177

[jfb70604-bib-0015] da Silva, N. G. , Ratko, J. , Corrêa, A. P. N. , da Silva, D. O. , Herrerias, T. , Pereira, D. M. C. , Schleger, I. C. , Neundorf, A. K. A. , de Souza, M. R. D. P. , & Donatti, L. (2024). Physiological strategies of acute thermal conditions of *Rhamdia voulezi* collected in the Iguaçu river watershed, Paraná, Brazil: Biochemical markers of metabolic and oxidative stress. Environmental Science and Pollution Research, 31, 37681–37704. 10.1007/s11356-024-33718-8 38780841

[jfb70604-bib-0016] Dalvi, R. S. , Das, T. , Debnath, D. , Yengkokpam, S. , Baruah, K. , Tiwari, L. R. , & Pal, A. K. (2017). Metabolic and cellular stress responses of catfish, *Horabagrus brachysoma* (Günther) acclimated to increasing temperatures. Journal of Thermal Biology, 65, 32–40. 10.1016/j.jtherbio.2017.02.003 28343573

[jfb70604-bib-0017] Dawood, M. A. O. , Alkafafy, M. , & Sewilam, H. (2022). The antioxidant responses of gills, intestines and livers and blood immunity of common carp (*Cyprinus carpio*) exposed to salinity and temperature stressors. Fish Physiology and Biochemistry, 48, 397–408. 10.1007/s10695-022-01052-w PMC9005402 35171388

[jfb70604-bib-0018] Dettleff, P. , Zuloaga, R. , Fuentes, M. , Gonzalez, P. , Aedo, J. , Estrada, J. M. , Molina, A. , & Valdés, J. A. (2020). Physiological and molecular responses to thermal stress in red cusk‐eel (*Genypterus chilensis*) juveniles reveals atrophy and oxidative damage in skeletal muscle. Journal of Thermal Biology, 94, 102750. 10.1016/j.jtherbio.2020.102750 33292991

[jfb70604-bib-0019] Devin, S. , Burgeot, T. , Giambérini, L. , Minguez, L. , & Pain‐Devin, S. (2014). The integrated biomarker response revisited: Optimization to avoid misuse. Environmental Science and Pollution Research, 21, 2448–2454. 10.1007/s11356-013-2169-9 24078236

[jfb70604-bib-0020] Donaldson, M. R. , Cooke, S. J. , Patterson, D. A. , & Macdonald, J. S. (2008). Cold shock and fish. Journal of Fish Biology, 73, 1491–1530. 10.1111/j.1095-8649.2008.02061.x

[jfb70604-bib-0022] dos Santos Silva, S. , de Morais Carvalho Ananias, I. , Magalhaes, T. B. , de Sena Souza, A. , dos Santos, F. A. C. , Melo, N. , Murgas, L. D. S. , Favero, G. C. , & Luz, R. K. (2024). Hematological, biochemical and oxidative responses induced by thermal shock in juvenile Tambaqui (*Colossoma macropomum*) and its hybrid Tambatinga (*Colossoma macropomum × Piaractus brachypomus*). Fish Physiology and Biochemistry, 50, 1079–1092. 10.1007/s10695-024-01321-w 38381279

[jfb70604-bib-0023] Duan, Y. , Li, H. , Li, J. , Bai, S. , Fu, S. , Zhou, Y. , Liu, S. , Li, R. , Liu, H. , Zhou, C. , & Lei, L. (2024). Integrated transcriptome and 16S rDNA analyses reveal that acute heat stress induces intestinal damage in *Gymnocypris eckloni* . Frontiers in Marine Science, 11, 1448313. 10.3389/fmars.2024.1448313

[jfb70604-bib-0024] Fagundes, D. B. , Santos, C. H. D. A. D. , Souza Nahum, É. M. S. D. , & Val, A. L. (2026). Transcriptional responses of the lower lip of *Colossoma macropomum*: An adaptation to aquatic surface respiration under hypoxic conditions. Comparative Biochemistry and Physiology. Part A, Molecular & Integrative Physiology, 319, 112025. 10.1016/j.cbpa.2026.112025 42242579

[jfb70604-bib-0025] Fan, J. , Ye, J. , Kamphorst, J. J. , Shlomi, T. , Thompson, C. B. , & Rabinowitz, J. D. (2014). Quantitative flux analysis reveals folate‐dependent NADPH production. Nature, 510, 298–302. 10.1038/nature13236 PMC4104482 24805240

[jfb70604-bib-0026] Fang, M. , Lei, Z. , Ruilin, M. , Jing, W. , & Leqiang, D. (2023). High temperature stress induced oxidative stress, gut inflammation and disordered metabolome and microbiome in tsinling lenok trout. Ecotoxicology and Environmental Safety, 266, 115607. 10.1016/j.ecoenv.2023.115607 37862746

[jfb70604-bib-0027] Figueiredo‐Fernandes, A. , Fontaínhas‐Fernandes, A. , Peixoto, F. , Rocha, E. , & Reis‐Henriques, M. A. (2006). Effects of gender and temperature on oxidative stress enzymes in Nile tilapia *Oreochromis niloticus* exposed to paraquat. Pesticide Biochemistry and Physiology, 85, 97–103. 10.1016/j.pestbp.2005.11.001

[jfb70604-bib-0029] Fu, B. , Zhou, D. , Qiu, X. , Zheng, J. , Yang, Q. , Xiao, Y. , Liao, S. , Li, Q. , & Xing, D. (2025). Mulberry leaf extract reduces damage by regulating the oxidative response, immune response and intestinal flora of largemouth bass (*Micropterus salmoides*) under chronic heat stress. Frontiers in Marine Science, 12, 1–17. 10.3389/fmars.2025.1543540

[jfb70604-bib-0030] Grim, J. M. , Miles, D. R. B. , & Crockett, E. L. (2010). Temperature acclimation alters oxidative capacities and composition of membrane lipids without influencing activities of enzymatic antioxidants or susceptibility to lipid peroxidation in fish muscle. The Journal of Experimental Biology, 213, 445–452. 10.1242/jeb.036939 PMC2808215 20086129

[jfb70604-bib-0031] Guderley, H. (2004). Metabolic responses to low temperature in fish muscle. Biological Reviews of the Cambridge Philosophical Society, 79, 409–427. 10.1017/s1464793103006328 15191230

[jfb70604-bib-0032] Guerreiro, I. , Castro, C. , Serra, C. R. , Coutinho, F. , Couto, A. , Peres, H. , Pousão‐Ferreira, P. , Corraze, G. , Oliva‐Teles, A. , & Enes, P. (2023). Feeding yellow Worms to meagre: Effects on whole‐body fatty acid profile and hepatic and intestine oxidative status. Antioxidants, 12, 1031. 10.3390/antiox12051031 PMC10215808 37237897

[jfb70604-bib-0033] Guillen, A. C. , Borges, M. E. , Herrerias, T. , Kandalski, P. K. , de Arruda Marins, E. , Viana, D. , de Souza, M. R. D. P. , Oliveira do Carmo Daloski, L. , & Donatti, L. (2019). Effect of gradual temperature increase on the carbohydrate energy metabolism responses of the Antarctic fish *Notothenia rossii* . Marine Environmental Research, 150, 104779. 10.1016/j.marenvres.2019.104779 31450038

[jfb70604-bib-0034] Günzler, W. A. , Steffens, G. J. , Grossmann, A. , Kim, S.‐M. A. , Ötting, F. , Wendel, A. , & Flohé, L. (1984). The amino‐acid sequence of bovine glutathione peroxidase. Hoppe‐Seyler's Zeitschrift für physiologische Chemie, 365, 195–212. 10.1515/bchm2.1984.365.1.195 6714945

[jfb70604-bib-0035] Habig, W. H. , Pabst, M. J. , Fleischner, G. , Gatmaitan, Z. , Arias, I. M. , & Jakoby, W. B. (1974). The identity of glutathione S‐transferase B with ligandin, a major binding protein of liver. Proceedings of the National Academy of Sciences, 71, 3879–3882. 10.1073/pnas.71.10.3879 PMC434288 4139704

[jfb70604-bib-0036] Hilsdorf, A. W. S. , Hallerman, E. , Valladão, G. M. R. , Zaminhan‐Hassemer, M. , Hashimoto, D. T. , Dairiki, J. K. , Takahashi, L. S. , Albergaria, F. C. , Gomes, M. E. d. S. , Venturieri, R. L. L. , Moreira, R. G. , & Cyrino, J. E. P. (2022). The farming and husbandry of *Colossoma macropomum*: From Amazonian waters to sustainable production. Reviews in Aquaculture, 14, 993–1027. 10.1111/raq.12638

[jfb70604-bib-0037] Islam, M. J. , Kunzmann, A. , & Slater, M. J. (2021). Extreme winter cold‐induced osmoregulatory, metabolic, and physiological responses in European seabass (*Dicentrarchus labrax*) acclimatized at different salinities. Science of the Total Environment, 771, 145202. 10.1016/j.scitotenv.2021.145202 33736134

[jfb70604-bib-0038] Johnston, I. A. , Bower, N. I. , & Macqueen, D. J. (2011). Growth and the regulation of myotomal muscle mass in teleost fish. The Journal of Experimental Biology, 214, 1617–1628. 10.1242/jeb.038620 21525308

[jfb70604-bib-0039] Joy, S. , Alikunju, A. P. , Jose, J. , Sudha, H. S. H. , Parambath, P. M. , Puthiyedathu, S. T. , & Philip, B. (2017). Oxidative stress and antioxidant defense responses of *Etroplus suratensis* to acute temperature fluctuations. Journal of Thermal Biology, 70, 20–26. 10.1016/j.jtherbio.2017.10.010 29108554

[jfb70604-bib-0040] Kammer, A. R. , Orczewska, J. I. , & O'Brien, K. M. (2011). Oxidative stress is transient and tissue specific during cold acclimation of threespine stickleback. Journal of Experimental Biology, 214, 1248–1256. 10.1242/jeb.053207 21430200

[jfb70604-bib-0041] Laiz‐Carrión, R. , Sangiao‐Alvarellos, S. , Guzmán, J. M. , Martín del Río, M. P. , Míguez, J. M. , Soengas, J. L. , & Mancera, J. M. (2002). Energy metabolism in fish tissues related to osmoregulation and cortisol action. Fish Physiology and Biochemistry, 27, 179–188. 10.1023/B:FISH.0000032725.96481.b8

[jfb70604-bib-0042] Li, L. , Liu, Z. , Quan, J. , Sun, J. , Lu, J. , & Zhao, G. (2023). Dietary nano‐selenium alleviates heat stress‐induced intestinal damage through affecting intestinal antioxidant capacity and microbiota in rainbow trout (O*ncorhynchus mykiss*). Fish & Shellfish Immunology, 133, 108537. 10.1016/j.fsi.2023.108537 36639066

[jfb70604-bib-0043] Li, Q. , Xiong, L. , Zhu, Y. , Zheng, A. , & Zheng, S. (2024). Effects of acute temperature stress on the expression of related genes in the brain of *Opsariichthys bidens* . Fishes, 9, 248. 10.3390/fishes9070248

[jfb70604-bib-0044] Li, S. , Wang, R. , Dai, Z. , Wang, C. , & Wu, Z. (2021). Dietary supplementation with Yucca schidigera extract alleviated heat stress‐induced unfolded protein response and oxidative stress in the intestine of Nile tilapia (*Oreochromis niloticus*). Ecotoxicology and Environmental Safety, 219, 112299. 10.1016/j.ecoenv.2021.112299 33993089

[jfb70604-bib-0045] Liu, Y. , Mao, X. , Song, K. , Wang, L. , Li, X. , Zhang, C. , Lu, K. , & Rahimnejad, S. (2025). Dietary tauroursodeoxycholic acid supports growth and gut health in spotted seabass (*Lateolabrax maculatus*) under thermal stress conditions. Fish & Shellfish Immunology, 163, 110423. 10.1016/j.fsi.2025.110423 40381925

[jfb70604-bib-0046] Lu, D.‐L. , Ma, Q. , Sun, S.‐X. , Zhang, H. , Chen, L.‐Q. , Zhang, M.‐L. , & Du, Z.‐Y. (2019). Reduced oxidative stress increases acute cold stress tolerance in zebrafish. Comparative Biochemistry and Physiology Part A: Molecular & Integrative Physiology, 235, 166–173. 10.1016/j.cbpa.2019.06.009 31220619

[jfb70604-bib-0047] Lushchak, V. I. (2011). Environmentally induced oxidative stress in aquatic animals. Aquatic Toxicology, 101, 13–30. 10.1016/j.aquatox.2010.10.006 21074869

[jfb70604-bib-0048] Lushchak, V. I. , & Bagnyukova, T. V. (2006a). Temperature increase results in oxidative stress in goldfish tissues. 2. Antioxidant and associated enzymes. Comparative Biochemistry and Physiology Part C: Toxicology & Pharmacology, 143, 36–41. 10.1016/j.cbpc.2005.11.018 16426898

[jfb70604-bib-0049] Lushchak, V. I. , & Bagnyukova, T. V. (2006b). Temperature increase results in oxidative stress in goldfish tissues. 1. Indices of oxidative stress. Comparative Biochemistry and Physiology Part C: Toxicology & Pharmacology, 143, 30–35. 10.1016/j.cbpc.2005.11.017 16423562

[jfb70604-bib-0050] Lv, H. , Liu, Y. , Li, H. , Yin, X. , Wang, P. , Qu, X. , Gao, Y. , Li, W. , & Chu, Z. (2021). Modulation of antioxidant enzymes, heat shock protein, and intestinal microbiota of large yellow croaker (*Larimichthys crocea*) under acute cold stress. Frontiers in Marine Science, 8, 1–10. 10.3389/fmars.2021.725899

[jfb70604-bib-0051] Madesh, M. , & Balasubramanian, K. A. (1997). Activation of liver mitochondrial phospholipase A_2_ by superoxide. Archives of Biochemistry and Biophysics, 346, 187–192. 10.1006/abbi.1997.0288 9343365

[jfb70604-bib-0052] Magouz, F. , Mahmoud, S. , Moustafa, E. M. M. , & ElMorsy, R. (2022). Effectiveness of Allzyme SSF and Polizyme® multimix feed additives on growth performance, feed utilization and immunological parameters of Nile tilapia (*Oreochromis niloticus*) fingerlings. Journal of the Hellenic Veterinary Medical Society, 73, 3941–3950. 10.12681/jhvms.23567

[jfb70604-bib-0053] Mailloux, R. J. (2018). Mitochondrial antioxidants and the maintenance of cellular hydrogen peroxide levels. Oxidative Medicine and Cellular Longevity, 2018, 7857251. 10.1155/2018/7857251 PMC6051038 30057684

[jfb70604-bib-0054] Malek, R. L. , Sajadi, H. , Abraham, J. , Grundy, M. A. , & Gerhard, G. S. (2004). The effects of temperature reduction on gene expression and oxidative stress in skeletal muscle from adult zebrafish. Comparative Biochemistry and Physiology Part C: Toxicology & Pharmacology, 138, 363–373. 10.1016/j.cca.2004.08.014 15533794

[jfb70604-bib-0055] Melo, N. , de Souza, S. P. , Konig, I. , de Jesus Paula, D. A. , Ferreira, I. S. , Luz, R. K. , & Murgas, L. D. S. (2024). Sensitivity of different organs and tissues as biomarkers of oxidative stress in juvenile tambaqui (*Colossoma macropomum*) submitted to fasting. Comparative Biochemistry and Physiology Part A: Molecular & Integrative Physiology, 291, 111595. 10.1016/j.cbpa.2024.111595 38316170

[jfb70604-bib-0085] Meurer, S. , & Filho, E. Z. (1997). Limitações e potencialidades do cultivo de tambaqui (Colossoma macropomum Cuvier, 1818) na região subtropical brasileira. Boletim do Instituto de Pesca, 24(Especial), 169–172.

[jfb70604-bib-0056] Michie, L. , Thiem, J. , Boys, C. , & Mitrovic, S. (2020). The effects of cold shock on freshwater fish larvae and early‐stage juveniles: Implications for river management. Conservation Physiology, 8, 1–10. 10.1093/conphys/coaa092 PMC7566967 33093957

[jfb70604-bib-0057] Papoutsoglou, S. E. (2012). The role of the brain in farmed fish. Reviews in Aquaculture, 4, 1–10. 10.1111/j.1753-5131.2012.01056.x

[jfb70604-bib-0058] Paulino, R. R. , Pereira, R. T. , Diógenes, A. , de Oliveira, K. R. B. , Oliveira, D. C. F. , Peres, H. , Oliva‐Teles, A. , & Rosa, P. V. (2026). Effect of dietary oil source on mitigating oxidative stress in tambaqui (*Colossoma macropomum*) juveniles subjected to acute cold stress. Aquaculture International, 34, 68. 10.1007/s10499-026-02463-9

[jfb70604-bib-0059] Pereira, D. M. C. , Resende, A. C. , Schleger, I. C. , Neundorf, A. K. A. , Romão, S. , de Souza, M. R. D. P. , Herrerias, T. , & Donatti, L. (2023). Integrated biomarker response index as an ally in the observation of metabolic biomarkers in muscle of *Astyanax lacustris* exposed to thermal variation. Biochimie, 210, 3–13. 10.1016/j.biochi.2022.10.010 36273764

[jfb70604-bib-0060] Qi, Z.‐H. , Liu, Y.‐F. , Luo, S.‐W. , Chen, C.‐X. , Liu, Y. , & Wang, W.‐N. (2013). Molecular cloning, characterization and expression analysis of tumor suppressor protein p53 from orange‐spotted grouper, *Epinephelus coioides* in response to temperature stress. Fish & Shellfish Immunology, 35, 1466–1476. 10.1016/j.fsi.2013.08.011 24012751

[jfb70604-bib-0061] Ramos‐Oliveira, C. , Magalhães, R. , Serra, C. , Belo, I. , Oliva‐Teles, A. , & Peres, H. (2026). Comparative effects of untreated and high‐solid loading pre‐treated *Codium tomentosum* on oxidative and immune responses in European seabass (*Dicentrarchus labrax*). Fishes, 11, 151. 10.3390/fishes11030151

[jfb70604-bib-0062] Ratko, J. , Gonçalves da Silva, N. , Ortiz da Silva, D. , Paula Nascimento Corrêa, A. , Mauro Carneiro Pereira, D. , Cristina Schleger, I. , Karla Alves Neundorf, A. , Herrerias, T. , Rita Corso, C. , Rosa Dmengeon Pedreiro de Souza, M. , & Donatti, L. (2022). Can high‐ and low‐temperature thermal stress modulate the antioxidant defense response of *Astyanax lacustris* brain? Brain Research, 1797, 148118. 10.1016/j.brainres.2022.148118 36240883

[jfb70604-bib-0063] Refaey, M. M. , Mehrim, A. I. , El‐Komy, M. M. , Zenhom, O. A. , & Mansour, A. T. (2023). Chronic cold‐stress induced histopathological changes, oxidative stress, and alterations in liver functions and nutrient composition of hybrid red tilapia and the potential protection of unsaturated fatty acids. Frontiers in Marine Science, 10, 1–14. 10.3389/fmars.2023.1148978

[jfb70604-bib-0064] Reid, C. H. , Patrick, P. H. , Rytwinski, T. , Taylor, J. J. , Willmore, W. G. , Reesor, B. , & Cooke, S. J. (2022). An updated review of cold shock and cold stress in fish. Journal of Fish Biology, 100, 1102–1137. 10.1111/jfb.15037 35285021

[jfb70604-bib-0065] Reznick, A. Z. , & Packer, L. (1994). Oxidative damage to proteins: Spectrophotometric method for carbonyl assay. In L. Packer (Ed.), Methods in enzymology, oxygen radicals in biological systems part C (pp. 357–363). Academic Press. 10.1016/S0076-6879(94)33041-7 8015470

[jfb70604-bib-0066] Rossi, A. , Bacchetta, C. , & Cazenave, J. (2017). Effect of thermal stress on metabolic and oxidative stress biomarkers of *Hoplosternum littorale* (Teleostei, Callichthyidae). Ecological Indicators, 79, 361–370. 10.1016/j.ecolind.2017.04.042

[jfb70604-bib-0067] Roychowdhury, P. , Aftabuddin, M. , & Pati, M. K. (2021). Thermal stress–induced oxidative damages in the liver and associated death in fish, *Labeo rohita* . Fish Physiology and Biochemistry, 47, 21–32. 10.1007/s10695-020-00880-y 33058003

[jfb70604-bib-0068] Sanchez, W. , Burgeot, T. , & Porcher, J.‐M. (2013). A novel “Integrated Biomarker Response” calculation based on reference deviation concept. Environmental Science and Pollution Research, 20, 2721–2725. 10.1007/s11356-012-1359-1 23208755

[jfb70604-bib-0069] Sánchez‐Nuño, S. , Sanahuja, I. , Fernández‐Alacid, L. , Ordóñez‐Grande, B. , Fontanillas, R. , Fernández‐Borràs, J. , Blasco, J. , Carbonell, T. , & Ibarz, A. (2018). Redox challenge in a cultured temperate marine species during low temperature and temperature recovery. Frontiers in Physiology, 9, 923. 10.3389/fphys.2018.00923 PMC6056653 30065660

[jfb70604-bib-0070] Santos, F. A. C. , Boaventura, T. P. , da Costa Julio, G. S. , Cortezzi, P. P. , Figueiredo, L. G. , Favero, G. C. , Palheta, G. D. A. , de Melo, N. F. A. C. , & Luz, R. K. (2021). Growth performance and physiological parameters of *Colossoma macropomum* in a recirculating aquaculture system (RAS): Importance of stocking density and classification. Aquaculture, 534, 736274. 10.1016/j.aquaculture.2020.736274

[jfb70604-bib-0071] Schleger, I. C. , Pereira, D. M. C. , Resende, A. C. , Romão, S. , Herrerias, T. , Neundorf, A. K. A. , de Souza, M. R. D. P. , & Donatti, L. (2024). Metabolic responses in the gills of Yellowtail Lambari *Astyanax lacustris* under low‐ and high‐temperature thermal stress. Journal of Aquatic Animal Health, 36, 16–31. 10.1002/aah.10199 38217492

[jfb70604-bib-0072] Sun, J.‐L. , Zhao, L.‐L. , Liao, L. , Tang, X.‐H. , Cui, C. , Liu, Q. , He, K. , Ma, J.‐D. , Jin, L. , Yan, T. , Zhou, J. , & Yang, S. (2020). Interactive effect of thermal and hypoxia on largemouth bass (*Micropterus salmoides*) gill and liver: Aggravation of oxidative stress, inhibition of immunity and promotion of cell apoptosis. Fish & Shellfish Immunology, 98, 923–936. 10.1016/j.fsi.2019.11.056 31770642

[jfb70604-bib-0073] Szekeres, P. , Brownscombe, J. W. , Cull, F. , Danylchuk, A. J. , Shultz, A. D. , Suski, C. D. , Murchie, K. J. , & Cooke, S. J. (2014). Physiological and behavioural consequences of cold shock on bonefish (*Albula vulpes*) in The Bahamas. Journal of Experimental Marine Biology and Ecology, 459, 1–7. 10.1016/j.jembe.2014.05.003

[jfb70604-bib-0074] Toni, M. , Angiulli, E. , Miccoli, G. , Cioni, C. , Alleva, E. , Frabetti, F. , Pizzetti, F. , Grassi Scalvini, F. , Nonnis, S. , Negri, A. , Tedeschi, G. , & Maffioli, E. (2019). Environmental temperature variation affects brain protein expression and cognitive abilities in adult zebrafish (*Danio rerio*): A proteomic and behavioural study. Journal of Proteomics, 204, 103396. 10.1016/j.jprot.2019.103396 31150779

[jfb70604-bib-0075] Tseng, Y.‐C. , Chen, R.‐D. , Lucassen, M. , Schmidt, M. M. , Dringen, R. , Abele, D. , & Hwang, P.‐P. (2011). Exploring uncoupling proteins and antioxidant mechanisms under acute cold exposure in brains of fish. PLoS One, 6, e18180. 10.1371/journal.pone.0018180 PMC3064598 21464954

[jfb70604-bib-0076] Val, A. L. , & Almeida‐Val, V. M. F. (2026). Beyond the mean: Adaptive variability in Amazonian aquatic systems. Philosophical Transactions of the Royal Society of London. Series B, Biological Sciences, 381, 20250059. 10.1098/rstb.2025.0059 41852212

[jfb70604-bib-0077] Val, A. L. , & Wood, C. M. (2022). Global change and physiological challenges for fish of the Amazon today and in the near future. Journal of Experimental Biology, 225, jeb216440. 10.1242/jeb.216440 35582942

[jfb70604-bib-0078] Valladão, G. M. R. , Gallani, S. U. , & Pilarski, F. (2018). South American fish for continental aquaculture. Reviews in Aquaculture, 10, 351–369. 10.1111/raq.12164

[jfb70604-bib-0079] Volkoff, H. , & Rønnestad, I. (2020). Effects of temperature on feeding and digestive processes in fish. Temperature (Austin), 7, 307–320. 10.1080/23328940.2020.1765950 PMC7678922 33251280

[jfb70604-bib-0080] Wang, Y. , Li, C. , Pan, C. , Liu, E. , Zhao, X. , & Ling, Q. (2019). Alterations to transcriptomic profile, histopathology, and oxidative stress in liver of pikeperch (*Sander lucioperca*) under heat stress. Fish & Shellfish Immunology, 95, 659–669. 10.1016/j.fsi.2019.11.014 31706008

[jfb70604-bib-0081] Wen, B. , Jin, S.‐R. , Chen, Z.‐Z. , & Gao, J.‐Z. (2018). Physiological responses to cold stress in the gills of discus fish (*Symphysodon aequifasciatus*) revealed by conventional biochemical assays and GC‐TOF‐MS metabolomics. Science of the Total Environment, 640–641, 1372–1381. 10.1016/j.scitotenv.2018.05.401 30021304

[jfb70604-bib-0082] Woynárovich, A. , & Van Anrooy, R. (2019). Field guide to the culture of tambaqui (Colossoma macropomum, Cuvier, 1816). In Fisheries and Aquaculture Technical Paper no 624 Food and Agriculture Organization of the United Nations.

[jfb70604-bib-0083] Wu, S. M. , Liu, J. H. , Shu, L. H. , & Chen, C. H. (2015). Anti‐oxidative responses of zebrafish (*Danio rerio*) gill, liver and brain tissues upon acute cold shock. Comparative Biochemistry and Physiology Part A: Molecular & Integrative Physiology, 187, 202–213. 10.1016/j.cbpa.2015.05.016 26025641

[jfb70604-bib-0084] Zhou, C. , Yang, S. , Ka, W. , Gao, P. , Li, Y. , Long, R. , & Wang, J. (2022). Association of gut Microbiota with Metabolism in rainbow trout under acute heat stress. Frontiers in Microbiology, 13, 846336. 10.3389/fmicb.2022.846336 PMC9007319 35432278

