# Supplementary material for: Integrated multitissue analysis reveals distinct oxidative stress responses during recovery from cold shock in the Amazonian fish Colossoma macropomum
Source: J Fish Biol. 2026 Aug 10;109(4):2318–31. doi: 10.1111/jfb.70604 (PMC13675509; doi:10.1111/jfb.70604)
Supplement: Supplementary file 1 [file JFB-109-2318-s001.docx]

**Integrated multi-tissue analysis reveals distinct oxidative stress responses during recovery from cold shock in the Amazonian fish *Colossoma macropomum***

Naiara Melo^1^, Isaac Konig^2^, Stefania Priscilla de Souza^1^, Maria Fernanda Aquino Freitas^1^, Daniella Aparecida de Jesus Paula^3^, Ronald Kennedy Luz^4^, Priscila Vieira Rosa^1^, Luis David Solis Murgas^1^.

^1^Faculty of Animal Science and Veterinary Medicine, Federal University of Lavras, UFLA, Lavras, Minas Gerais, CEP 37200-900, Brazil

^2^Department of Biochemistry, Federal University of Rio Grande do Sul (UFRGS), Porto Alegre, Rio Grande do Sul, Brazil

^3^Federal University of Piauí, Professor Cinobelina Elvas Campus, 64900000, Bom Jesus, PI, Brazil

^4^Universidade Federal de Minas Gerais, Departamento de Zootecnia, Laboratório de Aquacultura, Avenida Antônio Carlos, 6627, zip code 30161-970, Belo Horizonte, Minas Gerais, Brazil

*Corresponding author: lsmurgas@ufla.br

**SUPPLEMENTAL METHODS**

1. Antioxidant Enzymatic Responses

Each tissue sample was initially weighed using an analytical balance and subsequently homogenized in ice-cold saline buffer (0.138 M NaCl; 0.0027 M KCl; pH 7.2) at a ratio of 1:10 (w/v). The homogenates were then centrifuged at 7,000 × g for 15 min at 4 °C. After centrifugation, the supernatant was carefully recovered, divided into four aliquots of 500 μL each, and stored at −80 °C until analysis. All assays were conducted in triplicate using a microplate reader, and the interval between tissue collection and completion of the biochemical analyses was approximately one week.

1.1. Superoxide dismutase activity

Superoxide dismutase (SOD; EC 1.15.1.1) activity was quantified following a modified version of the method described by Madesh and Balasubramanian (1997). In brief, 40 μL of tissue homogenate was added to 132 μL of phosphate-buffered saline (PBS) in a microplate well. Subsequently, 8 μL of MTT solution (0.6 g L⁻¹; 3-(4,5-dimethylthiazol-2-yl)-2,5-diphenyltetrazolium bromide) and 20 μL of pyrogallol (1.25 mM) were introduced to initiate the reaction. The plate was incubated at 37 °C for 5 min. The reaction was stopped by adding 150 μL of dimethyl sulfoxide (DMSO), and absorbance was measured at 570 nm in duplicate using a microplate reader. Negative controls consisted of reaction mixtures containing all reagents except pyrogallol and the sample. Total SOD activity was expressed as units (U) per milligram of protein, where one unit corresponds to the amount of enzyme required to produce 50% inhibition of superoxide radical–mediated reduction per minute.

1.2. Catalase activity

Catalase (CAT; EC 1.11.1.6) activity was determined following an adaptation of the method described by Aebi (1984), based on the spectrophotometric monitoring of hydrogen peroxide (H₂O₂) decomposition. Briefly, 10 μL of supernatant was first diluted in 90 μL of PBS (1:10 dilution). From this diluted sample, 10 μL was transferred to a microplate well containing 140 μL of 10 mM H₂O₂ solution to initiate the reaction. The decline in absorbance was measured at 240 nm every 10 s for 2 min, with all samples analyzed in triplicate. Enzyme activity was calculated and expressed as units (U) per milligram of protein.

1.3. Glutathione peroxidase activity

Glutathione peroxidase (GPx; EC 1.11.1.9) activity was determined according to the method of Günzler et al. (1984). The reaction mixture consisted of PBS (10 mL), 10 mM reduced glutathione (GSH; 2.6 mL), 1.6 mM NADPH (2.6 mL), 10 mM sodium azide (NaN₃; 2.6 mL), and 1 μL of glutathione reductase (GR; 0.5 U mL⁻¹). For the assay, 40 μL of tissue homogenate were added to each well of a microplate, followed by 200 μL of the reaction mixture. The reaction was initiated by adding 25 μL of 10 mM hydrogen peroxide. The decrease in absorbance was monitored at 340 nm, and GPx activity was calculated and expressed as units (U) per milligram of protein.

1.4. Glutathione reductase activity

Glutathione reductase (GR; EC 1.8.1.7) activity was determined following the methods described by Günzler et al. (1984). For the assay, 25 µL of homogenate was added to a 96-well microplate. Then, 200 µL of a reaction mixture composed of 8 mL PBS buffer, 2 mL GSSG solution, 2 mL NADPH solution, 4 mL DTPA (diethylenetriaminepentaacetic acid) solution, and 6 mL deionized water was also added. Enzymatic activity was measured kinetically at 340 nm, based on NADPH oxidation. GR activity was calculated and expressed as units (U) per milligram of protein.

1.5. Glutathione S-transferase activity

Glutathione S-transferase (GST; EC 2.5.1.18) activity was measured following the method described by Habig et al. (1974), using 1-chloro-2,4-dinitrobenzene (CDNB) and reduced glutathione (GSH) as substrates. Briefly, 25 μL of tissue homogenate was added to each microplate well, followed by 125 μL of reaction mixture prepared with 0.1 M CDNB in ethanol (380 μL), GSH solution (2.3 mL; 30.7 mg mL⁻¹), and PBS (12.32 mL). The enzymatic conjugation reaction was monitored at 340 nm by recording absorbance every 20 s for a total of 15 measurements. All samples were analyzed in duplicate using a microplate reader. Wells containing only PBS were used as negative controls. GST activity was calculated and expressed as units (U) per milligram of protein.

2. Assessment of tissue damage biomarkers
   2.1. Lipid peroxidation

Lipid peroxidation was evaluated using the TBARS assay as described by Buege and Aust (1978). For this procedure, 300 µL of tissue homogenate was combined with 200 µL of TBARS reagent. The TBARS reagent was prepared by dissolving 30 g of trichloroacetic acid (TCA) and 0.75 g of thiobarbituric acid (TBA) in 100 mL of deionized water, then adding 12.5 mL of 4 M HCl. The volume was then completed with an additional 100 mL of deionized water, and the solution was heated at 60 °C in a water bath until fully dissolved. After mixing the homogenate with the TBARS reagent, samples were vortexed and incubated at 37 °C for 15 min. Once cooled to room temperature, 420 µL of butyl alcohol was added. The mixture was vortexed again and centrifuged at 5,000 × g for 5 min. The resulting supernatant was transferred to 96-well flat-bottom microplates, and absorbance was measured at 532 nm. Lipid peroxidation levels were expressed as malondialdehyde (MDA) concentration (mg L⁻¹).

2.2. Protein carbonyl levels

Protein carbonyl (PCO) levels were quantified using the 2,4-dinitrophenylhydrazine (DNPH) derivatization method described by Reznick and Packer (1994). In brief, 200 µL of each homogenate was split into two tubes, designated as sample and control. The sample tube received 800 µL of DNPH solution, whereas the control tube was treated with 800 µL of 2.5 M HCl. Following a 1 h incubation at room temperature in the dark, proteins were precipitated with 20% trichloroacetic acid (TCA) and centrifuged at 10,000 × g for 10 min at 4 °C. The resulting pellets were washed once with 10% TCA and three times with an ethanol/ethyl acetate mixture (1:1, v/v). Pellets were then resuspended in 500 µL of guanidine hydrochloride and centrifuged to remove insoluble material. Supernatants were transferred in duplicate to a 96-well microplate, and absorbance was measured at 370 nm. PCO content was calculated from the difference between sample and control readings and expressed relative to total protein concentration.

2.3. Quantification of total protein levels

Total protein concentration in the homogenates was quantified as proposed by Bradford (1976). For the assay, 10 µL of the supernatant was added to 200 µL of Bradford reagent. Absorbance readings were taken at 595 nm in duplicate, using albumin for the calibration curve. The values were used to normalize the enzymatic assay data (SOD, CAT, GPx, GR, and GST) and the tissue damage biomarkers (MDA and PCO).

**References**

Aebi, H., 1984. Catalase in vitro. Methods Enzymol 105, 121–126. https://doi.org/10.1016/s0076-6879(84)05016-3

Buege, J.A., Aust, S.D., 1978. Microsomal lipid peroxidation. Methods Enzymol 52, 302–310. https://doi.org/10.1016/s0076-6879(78)52032-6

Flohé, L., Günzler, W.A., 1984. Assays of glutathione peroxidase. Methods Enzymol 105, 114–121. https://doi.org/10.1016/s0076-6879(84)05015-1

Günzler, W.A., Steffens, G.J., Grossmann, A., Kim, S.-M.A., Ötting, F., Wendel, A., Flohé, L., 1984. The Amino-Acid Sequence of Bovine Glutathione Peroxidase 365, 195–212. https://doi.org/10.1515/bchm2.1984.365.1.195

Habig, W.H., Pabst, M.J., Fleischner, G., Gatmaitan, Z., Arias, I.M., Jakoby, W.B., 1974. The Identity of Glutathione S-Transferase B with Ligandin, a Major Binding Protein of Liver. Proceedings of the National Academy of Sciences 71, 3879–3882. https://doi.org/10.1073/pnas.71.10.3879

Madesh, M., Balasubramanian, K.A., 1997. Activation of Liver Mitochondrial Phospholipase A2 by Superoxide. Archives of Biochemistry and Biophysics 346, 187–192. https://doi.org/10.1006/abbi.1997.0288

Reznick, A.Z., Packer, L., 1994. [38] Oxidative damage to proteins: Spectrophotometric method for carbonyl assay, in: Methods in Enzymology, Oxygen Radicals in Biological Systems Part C. Academic Press, pp. 357–363. https://doi.org/10.1016/S0076-6879(94)33041-7
